# Photo-Thermal Dry Reforming of Methane with PGM-Free and PGM-Based Catalysts: A Review

**DOI:** 10.3390/ma17153809

**Published:** 2024-08-01

**Authors:** Alessio Varotto, Umberto Pasqual Laverdura, Marta Feroci, Maria Luisa Grilli

**Affiliations:** 1 Energy Technologies and Renewable Sources Department, Italian National Agency for New Technologies, Energy and Sustainable Economic Development (ENEA), Via Anguillarese 301, 00123 Rome, Italy; umberto.pasqual@enea.it; 2 Department of Fundamental and Applied Sciences for Engineering (SBAI), Sapienza University of Rome, Via Castro Laurenziano, 7, 00161 Rome, Italy; marta.feroci@uniroma1.it

**Keywords:** photo-thermal, dry reforming of methane, platinum group metals (PGMs), PGM-based catalysts, PGM-free catalysts, syngas

## Abstract

Dry reforming of methane (DRM) is considered one of the most promising technologies for efficient greenhouse gas management thanks to the fact that through this reaction, it is possible to reduce CO_2_ and CH_4_ to obtain syngas, a mixture of H_2_ and CO, with a suitable ratio for the Fischer–Tropsch production of long-chain hydrocarbons. Two other main processes can yield H_2_ from CH_4_, i.e., Steam Reforming of Methane (SRM) and Partial Oxidation of Methane (POM), even though, not having CO_2_ as a reagent, they are considered less green. Recently, scientists’ challenge is to overcome the many drawbacks of DRM reactions, i.e., the use of precious metal-based catalysts, the high temperatures of the process, metal particle sintering and carbon deposition on the catalysts’ surfaces. To overcome these issues, one proposed solution is to implement photo-thermal dry reforming of methane in which irradiation with light is used in combination with heating to improve the efficiency of the process. In this paper, we review the work of several groups aiming to investigate the pivotal promoting role of light radiation in DRM. Focus is also placed on the catalysts’ design and the progress needed for bringing DRM to an industrial scale.

## 1. Introduction

**Figure 1:**
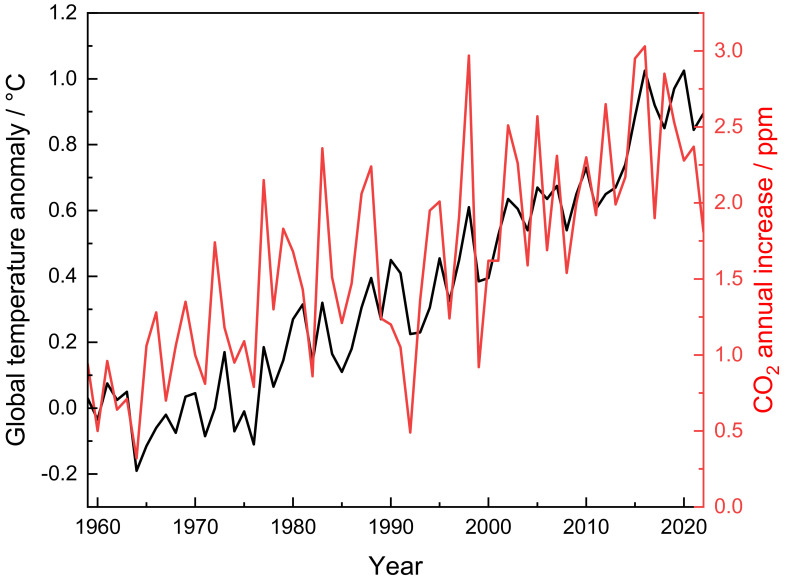
The link between increasing global temperature and increasing CO_2_ emissions from 1859 to 2022. The data are taken from ref. [5].

Currently, there is an increasing concentration of greenhouse gases (GHGs) in the atmosphere caused by anthropogenic activities, leading to global warming [1], especially due to CO_2_ and CH_4_. Correlations between the annual increase in temperature and GHGs have been analyzed over the years by many research centers, including NASA [2,3,4]. Figure 1 shows the chart of global temperature anomalies, which are deviations from a reference value or long-term average, and the increasing CO_2_ emissions in the period from 1959 to 2022. Despite the ten-year variation of some tenths of degrees, it must be considered that even 1 or 2 °C can have a huge impact on the global environment [5].

The escalating levels of CO_2_ and CH_4_ concentrations in the atmosphere have intensified concerns regarding climate change [6]. The Paris Agreement was signed by 196 parties who committed to reduce GHG emissions to limit global warming well below 2 °C above pre-industrial levels, increasing the ability to adapt to the adverse impacts of climate change and create forest climate resilience and low-GHG-emission development [7]. The target goals are to reduce CO_2_ emissions by at least 30 gigatons (${Gt}_{{CO}_{2}}$)/yr [8] using alternative energy sources and, at the same time, employing carbon capture, utilization and storage (CCUS), reducing costs and increasing energy efficiency. Europe must achieve these goals by 2030 to reduce GHG emissions, and this is part of the “Fit for 55” legislation that is part of the European Green Deal to achieve climate neutrality by 2050. During the last COP28 (Conference of the Parties) in Dubai, more than 200 parties discussed the world’s first “global stock-take” to ratchet up climate change action before the end of the decade, with the overarching aim to keep the global temperature increase below 1.5 °C. One of the most important points of this COP is that the agreement on mitigation measures now includes a fossil fuel phase-out instead of only a phase-down. This is the first COP in which fossil fuels were designated as the principal cause of climate change.

Dry reforming of methane (DRM) constitutes a valuable approach for effective CO_2_ and CH_4_ conversion to added-value syngas (H_2_ and CO), even though it is considered at present to be far from effectively mitigating the environmental issues related to GHG emissions because of the many challenges limiting its widespread application at the industrial scale.

Many review papers on dry reforming of methane reactions can be found in the literature and are focused on particular aspects, such as the catalysts’ active materials, the supports, the catalysts’ design, the catalysts’ deactivation by carbon deposition, etc. With this review paper on photo-thermal dry reforming of methane preceded by only a few previous ones [9,10,11,12,13,14,15,16], we aim to investigate the pivoting role of light irradiation focusing both on platinum group metal-free catalysts and catalysts containing platinum group metals as active elements.

This review will start with a description of the dry reforming of methane reaction, followed by a description of the other reactions forming syngas from methane (bi- and tri-reforming of methane). The principles of photo-thermal catalysis (PTC) will be introduced before the photo-thermal dry reforming of methane (PTC-DRM) description, with PTC-DRM being the focus of the present paper. Then, the catalysts for PTC-DRM, both containing platinum group metals (PGMs) and without PGMs, will be reviewed.

## 2. Dry Reforming of Methane (DRM) Reaction

**Figure 2:**
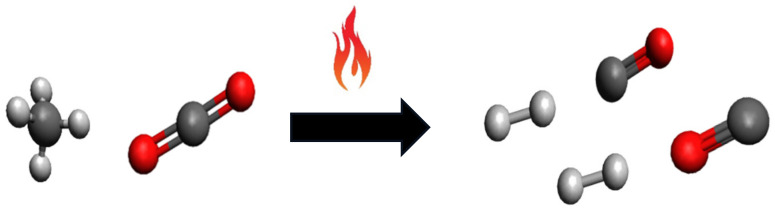
DRM reaction promoted by a source of heat.

In the dry reforming of methane, CO_2_ and CH_4_ react to obtain a syngas rich in CO and H_2_ [17]. The DRM reaction was studied for the first time by Fischer and Tropsch in 1928 using nickel and cobalt catalysts, and nowadays, it is proposed as a potential solution to convert CO_2_ on a large scale [8,18]. Equation (1) reports the DRM reaction and Figure 2 schematizes the DRM reaction promoted by a source of heat. The main characteristics of the DRM are that, according to the thermodynamics [19], it may yield a H_2_/CO ratio close to unity and is highly endothermic, so high temperatures (>650 °C) must be used to promote the DRM reaction, even in the presence of a catalyst [18].

(1) $${\mathrm{CH}}_{4}+{\mathrm{CO}}_{2}\;\to \;2\;H_{2}+2\;\mathrm{CO}\;\;{\Delta H}_{298\;K}^{0}=247\;kJ/mol$$

As the variation in enthalpy is positive, for Le Chatelier’s principle, high temperatures are needed to transform the reagents into syngas, as evident from the Gibbs Equation (2) [19,20,21]:

(2) $${\Delta G}_{f}^{0}={\Delta H}_{f}^{0}-{\Delta S}_{f}^{0}\times T$$

where ${\Delta H}_{f}^{0}$ is the variation in enthalpy formation, equal to 247 kJ/mol, and ${\Delta S}_{f}^{0}$ is the variation in entropy formation, equal to 0.26 kJ/(mol·K) [22]. ${\Delta G}_{f}^{0}$ is negative when T > 950 K [23].

It is important to underline that the H_2_/CO ratio in the DRM reaction is always lower than unity due to the reverse water gas shift (RWGS) equilibrium reaction (3):

(3) $${\mathrm{CO}}_{2}+H_{2}\;⇌\;\mathrm{CO}+H_{2}O\;{\Delta H}_{298\;K}^{0}=41\;kJ/mol$$

where part of the hydrogen formed during the DRM reaction reacts with carbon dioxide to form carbon monoxide and water [24]. Equations (1) and (3) are spontaneous when the temperature is above 650 °C, and this means that the RWGS is inevitable [25].

High temperatures are needed because of the high energies required to break the first C-H bond in methane (750 kJ/mol), which is the rate-determining step of the entire reaction, and the first C=O bond in carbon dioxide (476 kJ/mol).

**Figure 3:**
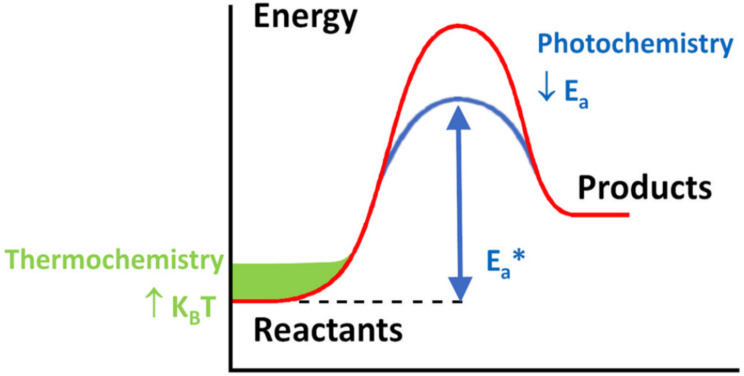
Photo-catalysis and thermo-catalysis contributions to PTC. During photo-catalysis, the activation energy barrier (Ea, red line) is reduced by the effect of light (Ea*, blue line), while the temperature effect is to provide additional thermal energy (kBT, green zone) to the energy path of an endothermic reaction. Figure reprinted with permission from [34]. Adapted from [35]. Copyright 2008 Royal Society of Chemistry.

To decrease the activation energies and thus the temperature needed to promote the DRM reaction, catalysts are generally used. The reaction can also be promoted by using external sources of light. Thus, as schematized in Figure 3, the DRM reaction can occur thermocatalytically (TC), photocatalytically (PC) and photothermocatalytically (PTC).

The first classical method employs heat to promote the DRM reaction, the second one uses a light source, typically a Xe or a Hg lamp or a concentrated sunlight, and in the third one, both heat and light are combined to increase the efficiency of the reaction. The last approach, i.e., the synergistic effect of light and heat, seems to be more powerful than the sum of its parts [26,27,28,29].

In the following sections, the fundamentals of photo-thermal (applying the principles of PC and TC) dry reforming of methane will be discussed [24,30,31,32,33]. Photo-thermal dry reforming of methane is superior compared to the other two pathways proposed; for thermocatalysis occurring at high temperatures (the reaction is endothermic), byproducts such as coke may be generated, and only an opportune catalyst design can mitigate the effect of catalyst deactivation by carbon deposition. At low temperatures, even if carbon deposition is reduced, to achieve high catalytic efficiencies, the assistance of a light source is necessary. On the other hand, a light source without heat assistance does not lead to high catalytic efficiency. Therefore, temperature plays a significant role in the DRM reaction, as reported by many authors. As an example, Sokefun et al. [33] have investigated the effect of Ru loading and reduction temperature of Ru-Ni-Mg/Ceria-Zirconia catalysts, achieving dry reforming activity between 450 and 510 °C, even though conversion efficiencies were lower in comparison to the values generally obtained at higher temperatures.

## 3. Bi- and Tri-Reforming of Methane

The DRM reaction converts the two GHGs CH_4_ and CO_2_ into syngas; however, two other main reactions can be used to convert methane into syngas: Steam Reforming of Methane (SRM) and Partial Oxidation of Methane (POM), which are reported in Equations (4) and (5):

(4) $${CH}_{4}+H_{2}O\to \;\mathrm{CO}+3H_{2}\;\;\Delta H_{298K}^{0}=206\;kJ/mol$$

(5) $${CH}_{4}+{0.5\;O}_{2}\to \;\mathrm{CO}+{2H}_{2}\;\;\Delta H_{298K}^{0}=-36\;kJ/mol$$

The SRM reaction provides syngas from methane and water, while the POM reaction uses oxygen in place of H_2_O [36].

When Equations (1) and (4) are combined together, in one step (6), the process is called bi-reforming of methane (BRM) or CSDRM (i.e., combined SRM and DRM). When Equations (1), (4) and (5) are combined together, in one step, the process is called tri-reforming of methane (TRM) (7).

(6) $${3CH}_{4}+{2H}_{2}O+{CO}_{2}\to \;4\mathrm{CO}+8H_{2}\;\;\Delta H_{298K}^{0}=206\;kJ/mol$$

(7) $${3CH}_{4}+{0.5\;O}_{2}+{CO}_{2}+H_{2}O\to \;4\mathrm{CO}+{7H}_{2}\;\;\Delta H_{298K}^{0}=104\;kJ/mol$$

BRM enhances the H_2_ production using SRM, due to the equilibrium in the water gas shift reaction. The combination of dry reforming with steam reforming leads to a syngas with flexible H_2_/CO ratios via easy adjustment of feed-stock composition and suitable for the Fisher–Tropsch production of methanol, oxo-alcohols and other chemicals. In addition, in BRM, carbon deposition on catalysts’ surface [37] is reduced.

Tahir et al. [38] synthesized a Co/HC_3_N_4_ catalyst with different Co loading and performed a photo-activity test using a Hg lamp with light intensity of 100 mW/cm^2^ at different feed mixtures of CH_4_/CO_2_. HC_3_N_4_ was used because of its ability to exploit solar energy, while cobalt was used to increase the active sites and capture the charge carriers. The highest production rates of H_2_ and CO were obtained in the case when 2% wt. of Co was used, reaching 689 and 95.8 µmol/g, respectively, after 4 h of irradiation time. In both DRM and BRM reactions, production rate decreased in the presence of water.

As in a DRM reaction, in a TRM reaction, oxides such as CeO_2_, ZrO_2_ and MgO are also generally used [39]. Due to the presence of CO_2_ as a reactant, support needs a high oxygen storage capacity, as it increases the conversion of carbon dioxide due to the basic character of these oxides [40]. Oxide supports are chosen due to their high thermal stability and their high oxygen storage capacity, and it is even possible to use them as photocatalysts for one of these reactions.

Despite BRM and TRM showing very promising potential because of their higher H_2_/CO ratios and reduced carbon deposition on catalysts’ surface, several challenges, as in DRM, exist for their scalability [37].

**Figure 4:**
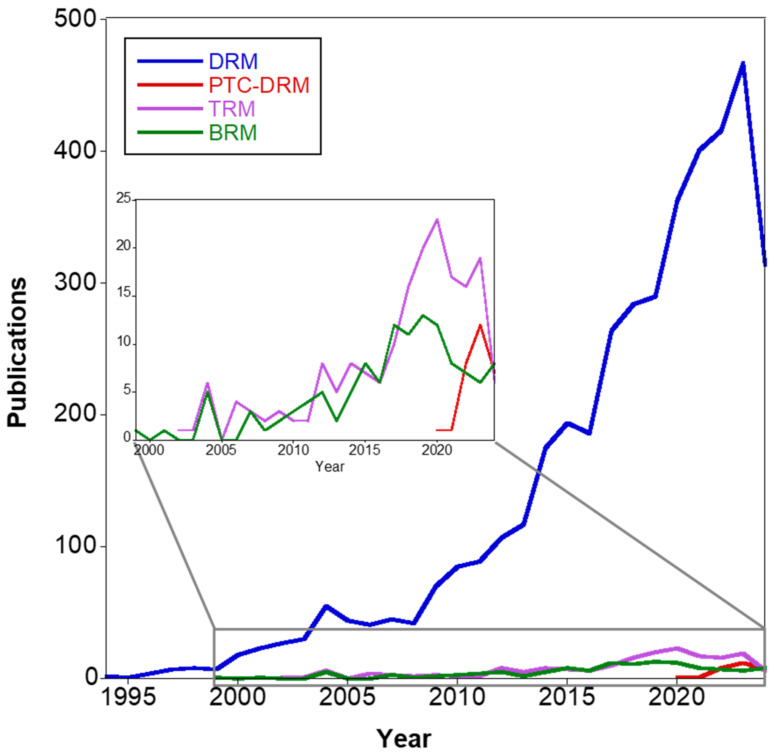
Publications trends over the years of “DRM”, “PTC-DRM”, “BRM” and “TDRM” (source: Scopus: 5 July 2024).

Figure 4 reports the trends of publications about DRM, PTC-DRM, BRM and TRM starting from 1994, and ref. [41] reports a bibliometric study on dry reforming of methane.

## 4. Fundamentals of Photo-Thermal Catalysis

In this section, the focus is on the fundamentals of Photo-Thermal Catalysis (PTC) to analyze how catalysts’ performances are improved by light absorption. In PTC, absorption of photons by a material promotes electrons from the valence to the conduction band and generates electron–hole pairs, which will participate in the subsequent redox reactions.

PTC involves the interaction between photons, electrons and phonons, giving rise to photo-thermal and/or Localized Surface Plasmon Resonance (LSPR) effects [42,43].

**Figure 5:**
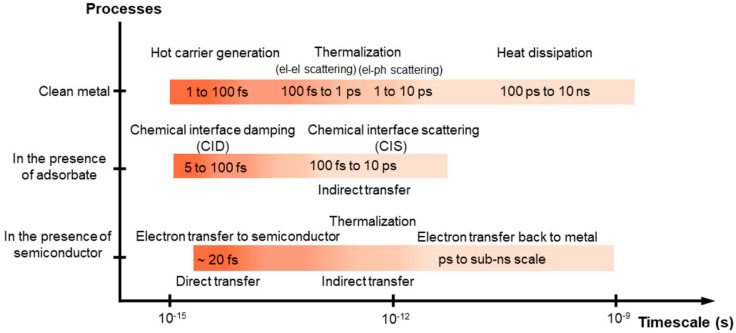
Time scales of plasmon-induced hot carrier generation, hot electron transfer, and thermalization processes. Figure reprinted with permission from [46].

While PTC utilizes lattice vibrations induced by light to achieve the photo-thermal conversion, the LSPR mechanism generates an electric field enhancement by plasmons and it utilizes electron dispersion to generate hot carriers, i.e., charge carriers (electrons) that dissipate energy in the form of heat. The LSPR mechanism occurs when resonant conditions are satisfied, i.e., when the energy of the external source of light matches the work function of the metal nanoparticles on the surface. In this way, resonant frequencies of conduction electrons and incident electromagnetic waves are enhanced on the surface of plasmonic nanoparticles, in so-called hot spots. The excess energy can decay through several pathways, radiative or non-radiative. In non-radiative decay, electrons will generate hot charge carriers in the plasmonic structure through intra- (s-to-s transitions) or interband (d-to-s transitions) mechanisms (this phenomenon is called “Landau damping”). In this case, electron–hole pair excitations and electron–electron collisions occur. To facilitate the promotion of electrons from the valence band to the conduction band, it is important to use materials with a narrow band gap. In fact, one of the main challenges in photo-catalysis is to avoid electron–hole recombination to facilitate the formation of products [44,45]. For plasmon-induced catalysis, several reaction mechanisms have been proposed on the basis of their time scale with or without an adsorbate or a semiconductor (Figure 5) [46].

**Figure 6:**
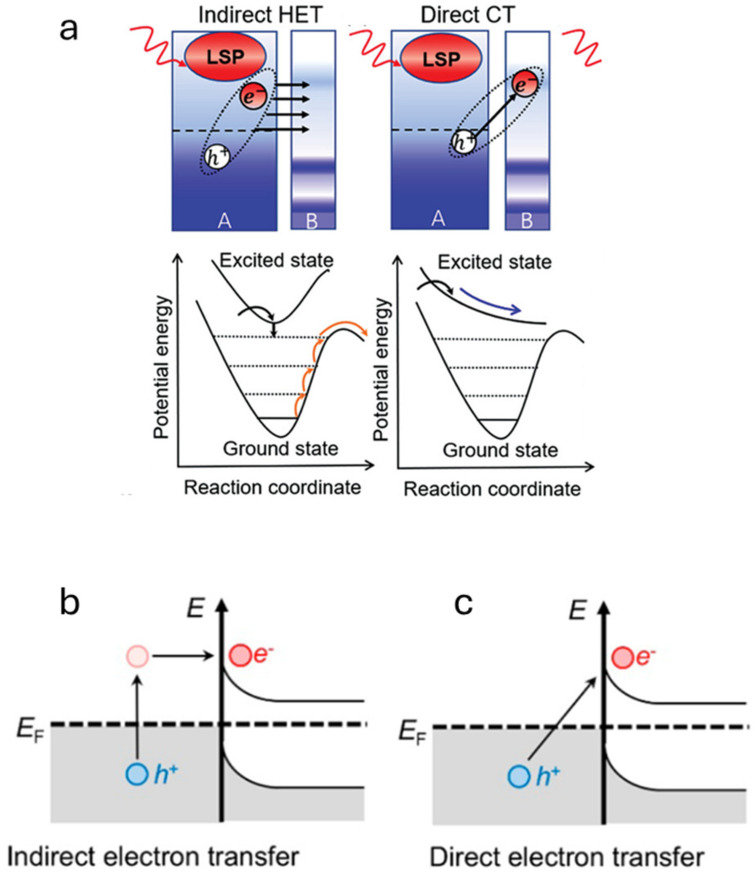
(**a**) Indirect and direct charge transfer mechanisms (HET—hot electron transfer; CT—charge transfer). A: metal, B: adsorbate. Figure modified from [49] (Creative Commons CC BY). (**b**) Indirect electron transfer and (**c**) direct electron transfer in a metal/semiconductor interface. Figures modified with permission from [46].

Indirect and direct hot electron transfer (HET) into a semiconductor is the topic of a very recent review [45]. In Figure 6, the indirect and direct charge transfer (CT) mechanisms are represented in the case of a plasmon-induced catalysis. The indirect electron transfer is a two-step process. In the first step, hot carriers (electrons and holes) are generated, while in the second one, there is the transfer of electrons and, depending on the existence of a single-component or a hetero-structured plasmonic photocatalyst, this can happen through a metal/adsorbate system (Figure 6a) or a metal/semiconductor system (Figure 6b,c). In the latter case, the electron transfer occurs through a Schottky barrier and the trapping of the transferred hot electrons increases their lifetime, because they do not travel back to the metal nanostructure.

The disadvantage of indirect electron transfer is the necessity to have electrons near the Fermi level. On the contrary, direct electron transfer is a single-step process, and it is assumed that energy loss is lower. However, high electric fields are needed for the direct electron transfer process to occur.

More insights about PTC are available in the literature [28,44,45,46,47,48,49,50].

In a reaction process involving light excitation, the efficiency η defined in (8) and the quantum efficiency Q.E. defined in (9) must be considered:

(8) $$\eta =\frac{(r_{CO}\times {\Delta_{c}H}_{CO}^{0}+r_{H_{2}}\times {\Delta_{c}H}_{H_{2}}^{0}-r_{{CH}_{4}}\times {\Delta_{c}H}_{{CH}_{4}}^{0})\;}{P_{illumination}}\times 100\%$$

where $P_{illumination}$ is the light power used, $r_{i}$ represents the reaction rate, Δ_c_$H_{i}^{0}$ is the standard combustion heat and i represents products and reagents, with Δ_c_$H_{{CO}_{2}}^{0}$ being equal to zero.

(9) $$Q.E.=\frac{\mathrm{the}\;\mathrm{number}\;\mathrm{of}\;\mathrm{reacted}\;\mathrm{electron}\;s\;(n)}{\mathrm{the}\;\mathrm{number}\;\mathrm{of}\;\mathrm{incident}\;\mathrm{photons}\;(N)}\times 100\%$$

The quantum efficiency Q.E. is the ratio of the reacted electrons and the number of incident photons N, which can be calculated using (10):

(10) $$N=\frac{I\times S\times t\times \lambda }{hc}$$

where I is the light power per area, S the surface area, t the time during which the catalyst is exposed to the light source, λ the wavelength, h the Planck’s constant and c the velocity of light [51].

In the next paragraph, the synergistic effect of light and heat on the DRM reaction will be discussed.

## 5. Photo-Thermal Dry Reforming of Methane

Solar-driven photo-thermal dry reforming of methane is considered among the most promising processes to convert GHGs into valuable chemicals. However, it suffers from some drawbacks that currently limit its application at the industrial scale, such as the high cost of electricity, the carbon tax, the limited number of hours during which natural sunlight is used to meet the energetic requirements of the reaction, the cost of the photo-catalyst and the catalyst lifetime [52,53,54,55]. Considering these issues [48], the requirements to obtain an efficient PTC-DRM are:

- high visible light absorption;
- good match between the band structure of the catalyst and the redox potentials of the reaction;
- good thermocatalytic activity.

**Figure 7:**
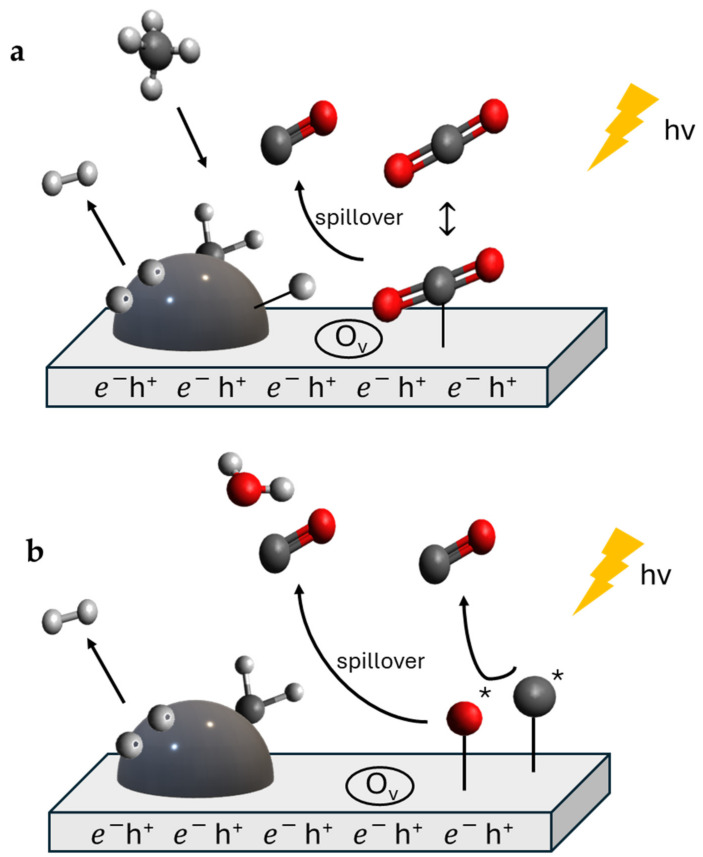
Simplified mechanism of PTC-DRM. (**a**) Activation of methane; (**b**) activation of carbon dioxide. The symbol * indicates the species adsorbed on the surface of the catalyst. O_v_ stands for oxygen vacancy.

In Figure 7, the description of a proposed simplified PTC-DRM mechanism is presented in which the principal steps are shown. Methane is dissociated by the active metal of the catalyst into CH_x_ and H* species, and H* species combine to produce H_2_ (Figure 7a). CO_2_ is activated by spillover on the catalyst’s surface (Figure 7b) and reacts with H* and CH* species to produce CO molecules and to release H_2_O, respectively. Methane activation is promoted by holes, and electrons promote the cleavage of the C-O bond in carbon dioxide.

In the literature, two main PTC mechanisms are proposed: monofunctional and bifunctional. It is hypothesized that in the monofunctional PTC mechanism, methane and carbon dioxide are activated on metal sites, following the typical Langmuir–Hinshelwood–Hougen–Watson mechanism; instead, in the bifunctional PTC mechanism, methane is activated on the metal sites and carbon dioxide on the surface of the support [56,57].

According to Langmuir–Hinshelwood–Hougen–Watson mechanism, in a bimolecular reaction such as DRM, three elementary steps are considered. The first one is the adsorption of the gases (A_(g)_ and B_(g)_) on adjacent sites, the second step is the reaction of molecules at the surface of the support and the third one is the desorption of the products (11):

A_(g)_ + B_(g)_ →  A_(ads)_ + B_(ads)_ →  P (11)

Light irradiation enhances, as heat, every step of the PTC mechanism, with the main differences that, compared to dark conditions, methane dissociation is faster [58].

In the study reported by Liu et al. [27], a catalyst with magnesium/aluminum-layered double hydroxides (Mg/Al-LDH) was used with Ni and Co alloys as active species. After light irradiation, methane decomposes into CH_x_ and H* species, and in a second step, C* and H* species are obtained before the desorption of the products. The activation energy required by using NiCo alloy was higher compared to that for catalysts employing only Ni or Co as active metals, to prevent the formation of carbon on the surface of the catalyst. This is one of the most accepted pathways for the DRM reaction; low activation energies of CH* oxidation compared to the oxidation of C* to CO* disfavor the deposition of carbon on the surface of catalysts.

Going deep into the DRM reaction, the value of the GHSV (Gas Hourly Space Velocity), i.e., the volume of a gas entering a reactor per hour per unit volume of catalyst, must also be considered. GHSV is calculated by the following Equation (12):

GHSV = [F_tot_·3600·(s·h^−1^)]/(V_cat_) (12)

where F_tot_ is the total flow rate used in the experiment and V_cat_ is the volume of the catalyst. GHSV is expressed as h^−1^. The inverse of this value is the space time τ (13), which represents the residence time of the gasses with catalyst:

τ = GHSV^−1^ (13)

The yield of the DRM reaction increases by increasing the residence time (decreasing the GHSV) due to the higher number of adsorbed reactants on the catalytic surface.

PTC is performed, usually, in a fixed bed reactor or in a stainless-steel reactor in which the catalyst is contained in a quartz tube to guarantee homogeneous heating. After the introduction of the sample into the reactor, the feed ratio of the gases is fixed at a certain temperature and the catalyst, if necessary, is reduced by hydrogen.

**Figure 8:**
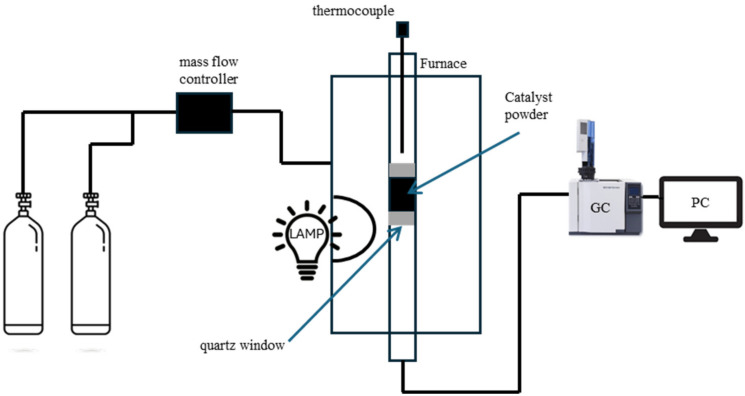
Exemplified scheme of a fixed bed reactor for PTC-DRM.

A simplified scheme of a type of reactor for PTC-DRM is presented in Figure 8.

In this representation, a reactor containing the catalyst is positioned inside a tubular vertical furnace and a source of light is used to promote the DRM reaction. After conversion, the produced gases are analyzed by a gas analyzer.

When the DRM is carried out at “low” temperatures, i.e., at T < 600 °C, the yields of the products are low and, in addition, at temperatures lower than 325 °C, the disproportion of carbon dioxide is favored. Disproportionation of CO_2_ produces different types of carbon species, which cause the deactivation of the catalysts [21,59]. On the other hand, starting from T > 650 °C, methane cracking is favored, especially on Ni-based catalysts. The formation of solid carbon C_(s)_ during CO_2_ reforming of CH_4_ may therefore occur either via CH_4_ cracking (14) or CO disproportionation (i.e., the Boudouard Reaction (15)) [24]:

(14) $${\mathrm{CH}}_{4}\;⇌\;2\;H_{2}\;+C_{\left(s\right)}\;\;{\Delta H}_{298K}^{0}=75\;kJ/mol$$

(15) $$2\mathrm{CO}\;⇌\;{\mathrm{CO}}_{2}\;+C_{\left(s\right)}\;\;{\Delta H}_{298K}^{0}=-172.5\;kJ/mol$$

**Figure 9:**
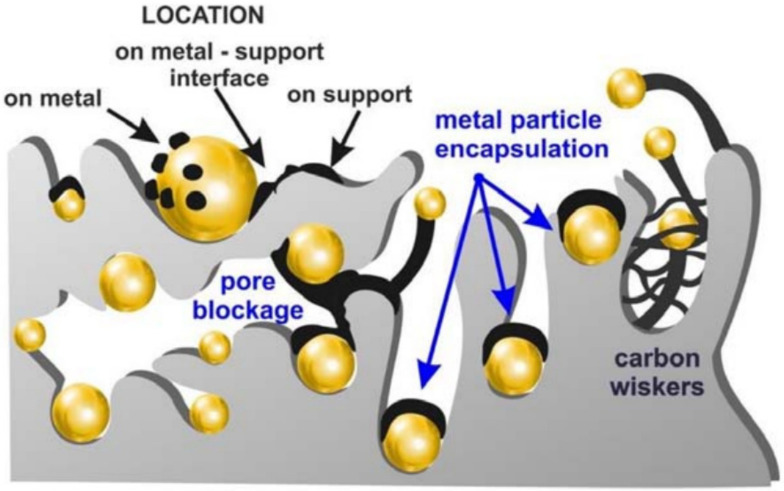
Formation of a coke deposit on a surface of a catalyst. Reprinted from [60]. Copyright MDPI 2021.

Figure 9 illustrates the formation of a coke deposit on a catalyst surface. It is evident how carbon deposits hinder the catalytic activity, blocking the active sites, which cannot be used by active metals [59].

Another crucial point is to understand how the carbon, derived from CO_2_ and CH_4_ adsorption, affects the lowering of the activation energies of the elementary steps [23]. In a recent review from Xu and Park [59], the main deactivation mechanisms of Ni-based catalysts are discussed, together with strategies to limit coke formation to improve the catalysts’ performance and stability.

## 6. Catalysts for PTC-DRM Reaction

In this last section, catalysts for the DRM reaction have been divided into two main classes: PGM-based and PGM-free catalysts, according to the active metal components.

As already said, PGM stands for Platinum Group Metal, i.e., platinum, palladium, rhodium, ruthenium, iridium and osmium. These metals have high catalytic activity due to their high heat and corrosion resistance, which make them almost unique for a vast range of industrial, medical and electronic applications. However, PGMs are listed among the critical raw materials (CRMs) for the EU [61], and many efforts are being devoted to their recovery from end-of-life products or to their partial or total substitution. It is worth noting that the support also plays a fundamental role in determining catalysts’ overall performance, because the support can provide additional catalytic sites, good dispersion of the active metal, increased surface area, etc., and the interaction between the support and the active component is fundamental in determining the catalytic activity and the selectivity of the catalysts [62].

### 6.1. PGM-Free Catalysts

The PGM-free catalysts for DRM are mostly based on Ni or Co as monometallic or bimetallic active compounds dispersed on different types of supports, mainly oxides [63,64,65,66]. It is worth nothing that Ni was recently added to the CRM list, while Co, which has been on the CRM list since 2011, presents additional cancerogenic issues [67]. Ni and Co cost is, however, much lower than that of PGMs [68].

In a recent study, Hu et al. [69] synthesized a catalyst for PTC-DRM containing Ni supported on Al_2_O_3_, which showed 142.8 and 160.2 mmol $g_{Ni}^{-1}$ h^−1^ yields of H_2_ and CO, respectively. Photo-thermal conditions helped in the formation of Ni^(0)^ and in obtaining hydrogen molecules starting from methane. However, catalysts supported on acidic materials, such as alumina, rapidly deactivate due to coke deposition, and this is one of the reasons why supports like CaO are often used [57]. In fact, CaO is a promising support for the DRM reaction, because of its high coke resistance, good dispersion of metal and strong metal–support interaction. Moreover, it is cheap, easily available, non-corrosive and environmentally friendly [70]. CaO is used to provide basic sites for the adsorption of CO_2_, but rarely, CaO is used alone, because it reacts with CO_2_ to form CaCO_3_. This is the reason why CaO is generally combined with other oxides to enhance the catalytic activity. ZrO_2_ is used with CaO to increase the activity of the catalyst, and it exhibits a strong metal–support interaction and stabilizes oxygen vacancies in the ZrO_x_ lattice. A very recent study on the modification of CaO-based adsorbents and Ni-based catalysts for DRM and CaL (calcium looping) was recently reported by Wang et al. [71]. Even SiO_2_ is an excellent choice because of its inertness, its low cost and low toxicity [72].

A catalyst with (Ni/CeO_2_)-SiO_2_ was demonstrated to be a useful obstacle to carbon deposition and sintering, due to its silica shell. From the Scherrer equation, the Ni sizes were 3.5 nm in the catalyst submitted to the DRM test. These values were close to the ones for the reduced catalyst, confirming the non-occurrence of Ni sintering. These findings were also confirmed by TEM images. Despite the fact that carbon deposition was confirmed by TGA analysis, its amount was very low, about 0.5% of the total amount of catalyst. The loss of carbon occurred in the temperature range of 800–1073 K and the reduction of nickel ion to metal was confirmed by XPS analysis. The fast CO_2_ activation was attributed to the reduction of Ce^4+^ to Ce^3+^, which led to a higher number of oxygen vacancies [73].

The creation of oxygen vacancies can be explained easily using cerium oxide as an example. Ce^4+^ leads to a re-distribution of the charge during the reduction of the ions; when CO_2_ molecules interact with the surface, the electronic density increases, CO_2_ accepts an electron and the adjacent oxygen vacancy acts as a sink to detach and accommodate the oxygen anion, replenishing the vacancy. At the end, the CO_2_ energy activation barrier for the breaking of the bond between C and O is reduced [55,74,75,76].

Perovskites are being extensively investigated for the DRM reaction and have been demonstrated to have high activity, even though challenges still exist for their application at the industrial scale due to carbon deposition and thermal stability. Perovskites are based on the general formula ABO_3_/A_2_BO_4_, where A is usually a rare earth, alkaline earth or alkali metal ion, and B is a transition metal cation. The catalytic properties of perovskites are significantly affected by the choice of A and B metal cations, by the preparation techniques and by the partial substitution of A and B cations. A recent review paper on dry reforming of methane over perovskite-derived catalysts is reported in [77]. As oxides, perovskites can handle high-temperature processes [57,78]. The insertion of calcium in a perovskite structure can improve oxygen storage capacity by creating oxygen defects, leading to a relatively high surface-to-volume ratio.

To study oxygen vacancies, two important techniques can be used: Electron Paramagnetic Resonance (EPR), in which, at a g value (specific value calculated for this technique) of 2.004, a high density of oxygen vacancies in the catalyst is evidenced, and X-ray Photoelectron Spectroscopy (XPS), by analyzing the O 1s region, where the three peaks around 530 eV can be attributed to oxygen lattice, vacancies and adsorbates. The oxygen vacancy-to-lattice oxygen ratio gives insights into the diffusion during the charge transfer [79,80,81].

Bimetallic alloys are very effective in enhancing the performances of the catalysts during heterogeneous catalysis. Zhang et al. [26] synthesized NiCo alloys on SiO_2_ supports and observed that CO_2_ conversion increased when light changed from high to low wavelengths, i.e., at higher energy. In their study, Field Emission Microscopy (FEM) was very useful to understand the role of hot carriers in PTC. 1.2 Ni_0.3_Co/SiO_2_ showed the highest values of quantum efficiency (Equation (9)) of 67 and 58% at 420 and 500 nm, respectively.

Takami et al. achieved conversion rates of 21% for CO_2_ and 20% for CH_4_ even at 473 K in a DRM reaction under visible light with a plasmonic Ni/Al_2_O_3_ photocatalyst. The production rates of CO and H_2_ were 1.87 and 1.20 mmol h^−1^, respectively [82].

Production rate can be calculated using (15) [51]:

(16) $$r_{i}={(C}_{outlet}\times V_{outlet}\times 60)/\left(22.4\times m_{cat}\right)$$

where $C_{outlet}$ and $V_{outlet}$ are, respectively, concentration and volume of the gas, $m_{cat}$ is the mass of the catalyst and $r_{i}$ is the outlet from the reactor.

In [28], Xie et al. compared three different experimental conditions, thermo-, photo- and photo-thermal, and used TiO_2_ as support, which is commonly used for photothermal catalysts. In their study, the authors reported how the synergistic effect of light and heat improves the formation rates of the products and the conversions of CO_2_ and CH_4_, mostly due to the separation of the excited electron/hole pairs.

Doping with alkaline metals or their oxide can increase the activity of catalysts due to their abilities to enhance the adsorption and activation of CO_2_ molecules. From the Arrhenius plot, apparent activation energy is reduced, passing from 57.2 kJ/mol under dark conditions to 29.5 kJ/mol under light conditions.

Fertout et al. [83] developed Ni catalysts on γ alumina doped with La_2_O_3_ and alkaline earth oxides (MgO, CaO and SrO), and they promoted the formation of carbonates, instead of hydroxides on these modified supports. Catalytic activity increased with the basicity of metals: Mg < Ca < Sr, probably because of a faster activation of CO_2_. Strontium has strong basicity, but magnesium improves the dispersion of Ni by surface rearrangement and calcium enhances the thermal stability and suppresses carbon deposition, even though it decreases the catalytic activity.

Khan et Tahir [84] developed TiO_2_ NP-embedded 2D Ti_3_C_2_ exfoliated sheets coupled with g-C_3_N_4_ to construct a 2D/2D g-C_3_N_4_/Ti_3_C_2_ heterojunction. The performance of the semiconductor was examined to analyze CO and H_2_ production under visible light. The graphitic carbon nitride (g-C_3_N_4_) after irradiation with an artificial light source acted as an electron donor. CO decomposition of methane was achieved by electrons donated by methane; holes, CH_x_ species and H^+^ ions reacted with methane to form a H_2_ molecule.

Tahir et al. [85] used g-C_3_N_4_ combined with Cu and observed that an increase in the amount of copper led to an increase of yields of the products. It is obvious from this study that the introduction of copper in the structure of graphitic carbon nitride is the key to obtain a H_2_/CO ratio close to unity. The catalyst’s structure was successful in promoting the DRM reaction because Cu/g-C_3_N_4_ decreased the CO selectivity due to a more prolific production of electrons.

Table 1 reports the DRM performances of PGM-free catalysts on several types of supports. In Table 2, the performances of catalysts exposed to an external source of light are reported.

To better compare the different types of catalysts, the turnover frequency (T.O.F.) should also be considered. In [93], T.O.F. is expressed by two different Equations (17) and (18):

T.O.F. = specific rate (converted molecules·s^−1^·g^−1^)/L (active sites·g^−1^) (17)

T.O.F. = areal site (converted molecules·s^−1^·nm^−2^)/d_s_ (active sites·nm^−2^) (18)

Specific rate refers to converted molecules per second per mass of sample. L is expressed as active sites per mass of sample and d_s_ is expressed as active sites per surface area of sample (19).

(19) $$L=d_{s}\;(\mathrm{active}\;\mathrm{sites}/{\mathrm{nm}}^{2})\times A\;({\mathrm{nm}}^{2}/g)$$

where d_s_ is the surface density of active sites and A is the surface area of the catalyst.

The difficulty in calculating T.O.F. lies in the exact determination of active sites, which is why the definition of T.O.F. is still debated [94].

If T.O.F. increases, the conversions of reactants also increase, and this means that photo-thermal conditions are more efficient than thermal conditions. As an example, in [73], it was found that at the same temperature of 750 °C, in the presence of (Ni/CeO_2_)-SiO_2_ catalyst, the conversions of CO_2_ and CH_4_ using the photo-thermal approach were 42.8 and 19%, respectively, while using only the thermal approach, conversion rates were much lower, i.e., 10.5 and 6.4 for carbon dioxide and methane, respectively.

In the case of a structure-insensitive reaction, T.O.F. is constant. If the catalytic reaction is structure-sensitive, T.O.F. depends on the size of the catalyst particles [95].

In the work from Vogt et al. [96], the catalyst is formed by different-sized Ni nanoparticles on a SiO_2_ support to prevent the formation of carbon on its surface. By changing the morphology of the catalyst, two different conditions for SRM and DRM are found. In the first one, T.O.F. increases with the increase in Ni particle size. The explanation is that bonds are cleaved preferentially over highly under-coordinated atoms in the metal nanoparticles. A change in the morphology of the sample will contribute to a change in the value of T.O.F., which depends directly on the rate-determining step, and this is also related to the intermediates involved during the DRM reaction. As an example, FTIR experiments evidenced that there are peaks attributed to the presence of CO molecules adsorbed in two different positions, top and bridge, and the ratio of the two peaks corresponding to CO_ads-top_ and CO_ads-bridge_ is correlated with the T.O.F trend. 

In Table 3 the effect of different supports on Ni-based catalysts is reported.

### 6.2. PGM-Based Catalysts for Photo-Thermal DRM

In this section, performances of catalysts containing Platinum Group Metals as active metals for PTC-DRM will be discussed. These precious metals can improve radically the catalytic activity of the DRM reaction, and they have a strong resistance to carbon deposition [19,99].

However, as already mentioned, also for these catalysts the support plays a fundamental role in the photocatalytic response, as we can see in the next two examples.

Yao et al. [74] developed a highly efficient photothermal Rh/LaNiO_3_ catalyst for solar-driven DRM, obtaining high generation rates for H_2_ and CO of 452.3 and 527.26 mmol·g_Rh_^−1^·h^−1^, respectively, under the irradiation of a 300 W Xe lamp of 1.5 W/cm^2^, without external heating.

In a previous work from the same group, Yang et al. [100] developed an all-in-one photothermal and photoelectric catalytic DRM process employing a Rh/Ce_x_WO_3_ catalyst. Also, in this case, the experiment was carried out under irradiation of a Xe lamp without external heating. For 1.8 W/cm^2^ irradiation, the generation rates of H_2_ and CO were 88.5 and 152.3 mmol·g_Rh_^−1^·h^−1^, respectively. 

In both studies, Rh was uniformly dispersed on the oxide supports and E_act_ (H_2_) and E_act_ (CO) were 43.89 and 39.99 kJ/mol for Rh/Ce_x_WO_3_ and 115.3 and 87.5 kJ/mol for Rh/LaNiO_3_ catalyst, respectively. In both situations, Rh provided a low electronic density and this led to the promotion of breaking of methane bonds and to the formation of H_2_. The formation of oxo-bridges lowered the start-up temperature of the DRM reaction, the temperature at which the reaction becomes spontaneous, according to Equation (2). In both studies, a migration of oxygen was reported, from Ce to W in the Rh/Ce_x_WO_3_ catalyst, and from La to Ni in the Rh/LaNiO_3_ catalyst. The weaker the binding in the oxo-bridge, the higher the migration of oxygen atoms. This is the main reason why E_act_ was lower in the case that Ce_x_WO_3_ was used as a support. The higher production rates obtained in the case of a catalyst with LaNiO_3_ support were attributed to the high number of oxygen defects able to effectively activate CO_2_ and enhance the interaction between the support and the Rh nanoparticles.

Yin and coworkers [101], who used a catalyst made of Ru and Ni alloys on an Al_2_O_3_ support, obtained very high values of production rates of H_2_ and CO: 539 and 642 mmol·g^−1^·h^−1^, respectively. In their work, the light irradiation and the thermal decomposition of carbonates are used to promote the DRM reaction. Methane reacts with CO_2_ produced by thermal decomposition of MgCO_3_, producing MgO and syngas. MgCO_3_ decomposes completely into MgO at 400 °C in 5% CH_4_/Ar, as evident from the XRD analysis. Generation rates of H_2_ and CO increase during the first 20 min up to 0.68 and 0.44 mmol·g^−1^·h^−1^, respectively, and selectivity of carbon dioxide decreases by 20% after one hour with the consumption of MgCO_3_. The increase in generation rates is due to the formation of carbonates, external light irradiation and localized surface plasmon resonance effect.

Song et al. [102], who used a Pt-Au/SiO_2_ catalyst (Pt content= 0.5–1.5 wt.%), obtained H_2_ and CO generation rates of, respectively, 5.5 and 7.2 mmol·g^−1^h^−1^ at 400 °C using a light intensity of 0.6 μW·cm^−2^. This catalyst presents a high surface area of 436 m^2^/g and the aggregate structure made of Pt and Au is thermodynamically stable. The temperature is comparable to the one used for the case of Yin et al. [101], but there is a huge difference in the value of light intensity used in these two studies. In the study of Yin, in fact, the intensity of light (13.5 W/cm^2^) is much higher than that used in the study of Song (0.6 μW/cm^2^). This explains the difference in the values of generation rates of products.

In all of the above-mentioned studies, SEM analysis has evidenced that catalytic metals are well dispersed on each support and no agglomeration is found on the surface of the catalysts used.

One of the most used supports to perform PTC-DRM is anatase TiO_2_ due to its narrow band gap, its ability to suppress carbon deposition and its good redox properties for oxygen mobility [99].

Zhang et al. [58] have investigated the performances of a Pt/mesoporous-TiO_2_ catalyst under different experimental conditions: reaction temperature, intensity of light and CO_2_/CH_4_ molar ratio. Authors found that a moderate increase in CO_2_ partial pressure was beneficial to the further dissociation of CH_4_, while the total amount of dissociated H species diminished with CO_2_/CH_4_ greater than 1.08/1. For CO formation, the authors proposed two possible pathways; the first one consists of the decomposition of HCOO^-^ and the second one consists of the dissociation of the CO_2_ molecule. 

Wu et al. [103] reported two different situations for Pt nanocrystals partially confined in mesoporous CeO_2_ nanorods (Pt/CeO_2_-MNR). In the first one, under focused UV-Vis-IR illumination, r (H_2_) and r (CO) were, respectively, 5.7 and 6.0 mmol g^−1^ min^−1^, while under visible-IR illumination, r (H_2_) and r (CO) were 4.9 and 5.2 mmol g^−1^ min^−1^, respectively. In the latter case, authors found an improved photoactivation which inhibits CO disproportion as a major side reaction of coke formation, promoting the oxidation of carbon species produced by CH_4_ dissociation and thus increasing the H_2_/CO ratio.

Another study shows the effect of the structure of the support [104]. In this work, Pt is combined with two different supports, i.e., a mesoporous TiO_2_ and P25. Higher production rates are obtained in the first case because of the mesoporous structure, which shows a different light absorption response, a different recombination of electrons and holes and a narrower band gap. This explains why at the same temperature of 500 °C and at the same intensity of light of 4.76 W/cm^2^, the production rates of H_2_ and CO are 178.6 and 281 mmol·$g_{Pt}^{-1}$·h^−1^ and 39.8 and 105.6 mmol·$g_{Pt}^{-1}$·h^−1^ in the case of mesoporous titania and P25, respectively.

In the study from Zhang et al. [105], the photo-thermal properties of a Pt-Au/P25 composite catalyst have been enhanced with the LSPR effect. The authors found that plasmonic nanoparticles act as electron traps, inhibiting the recombination of electron–hole pairs and promoting the PTC-DRM reaction. In the case of Pt and Au, their behavior was identical due to their similar atomic number. Even if Pt-Au/P25 had the smallest value of surface area, the catalyst had a good catalytic activity due to the Fermi energy levels of both metals, which helped to lower the valence band (2.67 eV). This led to an increase in generation rates, which increased linearly with the light intensity increase, and to the promotion of a better migration of holes. In this study the Pt-Au/P25 catalyst had a strong metal–surface interaction, and no deactivation during a 10 h DRM test was observed. The DRM results show how light can improve yields of products, 85.38 and 201.92 mmol·$g_{Pt}^{-1}$·h^−1^ for H_2_ and CO, respectively, favoring the dissociation of methane and CO_2_, while lower values were obtained in the case that only heat was used, 27.60 mmol·$g_{Pt}^{-1}$·h^−1^ for H_2_ and 97 mmol·$g_{Pt}^{-1}$·h^−1^ for CO.

Platinum is a very efficient catalytic metal for the DRM reaction [32,80,106,107]. The DRM reaction can be better performed on the active sites of precious metals due to their higher electron cloud density being able to reinforce the redox reactions on the surface of the catalysts. By combining precious metals and metals such as nickel and cobalt, high yields of products can be achieved [64]. It can be noted that the sample “0.3 Ni”, reported in Table 4, has the highest value of BET surface area but deactivates very rapidly when compared to the other catalysts. Comparison of 0.2, 0.3 and 0.4 PdNi bimetallic catalysts shows that increasing the amount of Pd, two situations arise:

- the dispersion of Ni decreases;
- sintering becomes more significant, and this leads, inevitably, to a decrease in the BET surface area.

**Table 4:** BET results on different catalysts. Feed ratio of CH_4_/CO_2_ is 1/1.

Name Sample	Temperature °C	Light Intensity W/cm^2^	BET Surface Area m^2^/g	Pore Volume cm^3^/g	Average Pore Diameter nm	Catalytic Performances	References
P25	500	4.6	63.0	0.12	7.9	N.A.	[105]
Pt/P25	500	4.6	62.0	0.14	9.1	r (H_2_) = 65 mmol·g^−1^·h^−1^ r (CO) = 162 mmol·g^−1^·h^−1^ H_2_/CO = 0.40	[105]
Au/P25	500	4.6	60.0	0.14	9.3	N.A.	[105]
Pt-Au/P25	500	4.6	55.0	0.12	8.7	r (H_2_) = 86 mmol·g^−1^·h^−1^ r (CO) = 203 mmol·g^−1^·h^−1^ H_2_/CO = 0.42	[105]
mes-TiO_2_	500	3.8	91.8	0.25	10.7	N.A.	[104]
P25	500	3.8	62.0	0.12	7.5	N.A.	[104]
Pt/P25	500	3.8	54.3	0.11	8.4	r (H_2_) = 40 mmol·g^−1^·h^−1^ r (CO) = 106 mmol·g^−1^·h^−1^ H_2_/CO = 0.38	[104]
Ni/mes-TiO_2_	500	3.8	85.1	0.24	11.1	r (H_2_) = 31 mmol·g^−1^·h^−1^ r (CO) = 81 mmol·g^−1^·h^−1^ H_2_/CO = 0.38	[104]
Pt/mes-TiO_2_	500	3.8	77.5	0.22	11.4	r (H_2_) = 211 mmol·g^−1^·h^−1^ r (CO) = 309 mmol·g^−1^·h^−1^ H_2_/CO = 0.68	[104]
0.3 Ni	650	0	580.7	0.39	2.2	CH_4_ = 16% CO_2_ = 25% H_2_/CO = 0.69	[64]
0.2 PdNi	650	0	523.6	0.38	2.4	CH_4_ = 23% CO_2_ = 31% H_2_/CO = 0.83	[64]
0.3 PdNi	650	0	514.7	0.35	2.2	CH_4_ = 36% CO_2_ = 52% H_2_/CO = 0.81	[64]
0.4 PdNi	650	0	506.6	0.35	2.1	CH_4_ = 30% CO_2_ = 42% H_2_/CO = 0.81	[64]
Ni/CaO-Al_2_O_3_	700	0	76.1	0.21	11.1	CH_4 conv._ = 54% CO_2 conv._ = 59%	[81]
CeO_2_-Ni/CaO-Al_2_O_3_	700	0	90.7	0.29	13.2	CH_4 conv._ = 82% CO_2 conv._ = 84%	[81]

High surface area leads to a higher contact area for the reactants, leading to a higher activity. These last two examples underline the importance of the interaction between support and metal, as mentioned before, and it is evident in the two cases of Ni/CaO-Al_2_O_3_ and CeO_2_-Ni/CaO-Al_2_O_3_ that high surface areas resulted in high dispersion of Ni on the support, thus increasing the catalytic activity [81].

Moreover, as occurs in the case of PGM-free catalysts, an appropriate choice of support, doping and active metal is needed. On the one hand, in fact, doping of the support can induce localized states in the band gap, which may increase the absorption ability of the photocatalyst, but on the other hand, localized band states may increase the charge carrier recombination, decreasing the catalytic activity.

Table 5 reports the catalytic performances under different conditions for PGM-based catalysts.

In Table 6, the performance of catalysts for the photo-thermal dry reforming of methane reaction using different values of flow rates of feed gases is presented.

## 7. Conclusions

This paper presents an overview of the current trends in PGM-based and PGM-free catalysts for photo-thermal dry reforming of methane, also highlighting drawbacks for large-scale PTC-DRM applications. With appropriate catalyst design, dry reforming of methane at high temperatures produces a syngas with a high conversion efficiency, generally higher than that obtained in solar-driven DRM reactions. However, if on the one hand, DRM at high temperature does not meet the needs of further industrial applications due to the high costs of materials for the catalysts and reactor, then on the other hand, the limited utilization of visible light limits the scalability of PTC-DRM at the industrial level. The synergistic effect of heat and light irradiation can therefore be a valuable solution to lower the temperature of the DRM process and increase process yields, making PTC-DRM a possible competitive technology for converting greenhouse gases into fuels at a large scale. The yields of the DRM products may, in fact, be enhanced by the photoelectric effect, which leads to a decrease in activation energies under lower-temperature conditions. Supported precious metal are widely used in DRM; however, several non- precious metals show great potential for reducing DRM costs. A suitable combination of support and catalytic component is a key factor for enhancing the conversion efficiency of the PTC-DRM process; however, issues related to catalyst deactivation due to carbon deposition still need to be completely solved. Coke management is at present one of the main issues limiting the widespread application of DRM technology.

## Figures and Tables

**Table 1:** Gas hourly space velocity of state-of-the-art DRM catalysts at different temperatures and feed ratio CH_4_/CO_2_ equal to 1/1.

Catalysts	GHSV h^−1^	τ s	Temperature °C	Catalytic Performances	References
CeO_2_-Ni/CaO-Al_2_O_3_	9000	0.4	700	CH_4 conv_. = 81% CO_2 conv._ = 84%	[81]
Sm/Ni/Al_2_O_3_-CaO	12,000	0.3	700	CH_4 conv_. = 68% CO_2 conv._ = 43% H_2_/CO = 0.64	[86]
7%Ni/CaO-Al_2_O_3_	1800	0.5	700	CH_4 conv_. = 66% CO_2 conv._ =62% H_2_/CO = 0.85	[87]
Ni-CaO-ZrO_2_	48,000	0.075	750	CH_4 conv_. = 82% H_2_/CO = 0.94	[88]
CaZr_0.8_Ni_0.2_O_3-δ_	28,800	0.125	800	CH_4 conv_. = 95% CO_2 conv._ = 96% H_2_/CO = 0.98	[89]

**Table 2:** Catalytic performances of PGM-free catalysts. Production rates have been calculated using (16). For solar irradiation, a Xe lamp was used. Feed ratio of CH_4_/CO_2_ is 1/1.

Catalyst	Surface Temperature °C	Light Intensity mW·cm^−2^	Catalytic Performances	Reference
CN/TNT	25	20	r (H_2_) = 49 μmol·g^−1^·h^−1^ r (CO) = 75 μmol·g^−1^·h^−1^ H_2_/CO = 0.64	[84]
(Ni/CeO_2_)-SiO_2_	750	-	CH_4 conv._ = 66% CO_2 conv._ = 80% H_2_/CO = 0.90	[73]
Ni_3_Fe_1_ nanoalloy	350	3.62	r (H_2_) = 326 × 10^3^ μmol·g^−1^·h^−1^ r (CO) = 632 × 10^3^ μmol·g^−1^·h^−1^ H_2_/CO = 0.52	[90]
La/TiO_2_	100	150	r(H_2_) = 74 μmol·h^−1^·g_cat_^−1^ r (CO) = 183 μmol·h^−1^·g_cat_^−1^ H_2_/CO = 0.40	[91]
Cu-g-C_3_N_4_	100	100	r(H_2_) = 76 μmol·h^−1^·g_cat_^−1^ r(CO) = 142 μmol·h^−1^·g_cat_^−1^ H_2_/CO = 0.54	[85]
Ni/Ga_2_O_3_	391	1.9–3	r (H_2_) = 194 μmol·g^−1^·h^−1^ r (CO) = 206 μmol·g^−1^·h^−1^ H_2_/CO = 0.94	[92]

**Table 3:** Characteristics of Ni-based catalysts with different supports and coatings: turnover frequencies and conversion of methane and carbon dioxide. Feed ratio of CH_4_/CO_2_ is 1/1.

Samples	Temperature °C	T.O.F. (CH_4_) s^−1^	T.O.F. (CO_2_) s^−1^	CH_4conv_ %	CO_2conv_ %	References
Ni/SiO_2_@Al_2_O_3_	800	135.2	177.2	62.8	82.3	[97]
Ni/SiO_2_@MgO	800	120.0	171.1	50.0	71.3	[97]
Ni/SiO_2_@ZrO_2_	800	29.5	37.8	29.1	37.3	[97]
Ni/SiO_2_@TiO_2_	800	12.9	18.1	17.0	23.8	[97]
Ni-Zr/SiO_2_	400	0.32	0.32	2	2	[98]
Ni-Zr/SiO_2_	450	1.06	1.48	0.8	1.2	[98]
Ni-Si/ZrO_2_	400	0.50	0.44	4.3	3.8	[98]
Ni-Si/ZrO_2_	450	1.38	1.30	1.6	2.4	[98]

**Table 5:** Catalytic performances of PGM-based catalysts for photo-thermal dry reforming of methane under different working conditions. Xe lamp was used, except for [55], in which concentrated sunlight was used. Feed ratio of CH_4_/CO_2_ is 1/1.

Catalyst	Surface Temperature °C	Light Intensity W·cm^−2^	Catalytic Performances	Reference
Rh/La_2_O_3_	340	1.5	r (H_2_) = 452 mmol·g^−1^·h^−1^ r (CO) = 527 mmol·g^−1^·h^−1^ H_2_/CO = 0.86	[74]
Pt/TiO_2_	500	4.67	r (H_2_) = 134 mmol·g^−1^·h^−1^ r (CO) = 221 mmol·g^−1^·h^−1^ H_2_/CO = 0.61	[58]
Pt/TiO_2_	350	4.67	r (H_2_) = 7 mmol·g^−1^·h^−1^ r (CO) = 22 mmol·g^−1^·h^−1^ H_2_/CO = 0.32	[58]
Pt/TiO_2_	500	3.72	r (H_2_) = 121 mmol·g^−1^·h^−1^ r (CO) = 200 mmol·g^−1^·h^−1^ H_2_/CO = 0.61	[58]
MgO/Pt/Zn-CeO_2_	600	3	r (H_2_) = 356 mmol·g^−1^·h^−1^ r (CO) = 516 mmol·g^−1^·h^−1^ H_2_/CO = 0.69	[55]
Ru-Al/LDH	350	13.5	r (H_2_) = 9 mmol·g^−1^·h^−1^ r (CO) = 11 mmol·g^−1^·h^−1^ H_2_/CO = 0.76	[101]
Pt/TiO_2_	500	0.4	r (H_2_) = 598 mmol·g^−1^·h^−1^ r (CO) = 902 mmol·g^−1^·h^−1^ H_2_/CO = 0.66	[51]
Pt/TiO_2_	700	0.4	r (H_2_) = 1103 mmol·g^−1^·h^−1^ r (CO) = 1495 mmol·g^−1^·h^−1^ H_2_/CO = 0.74	[51]
Pt/TiO_2_	500	3.76	r(H_2_) = 211 mmol·g^−1^·h^−1^ r(CO) = 309 mmol·g^−1^·h^−1^ H_2_/CO = 0.68	[104]
Pt/TiO_2_	500	2.2	r (H_2_) = 103 mmol·g^−1^·h^−1^ r (CO) = 179 mmol·g^−1^·h^−1^ H_2_/CO = 0.58	[104]

**Table 6:** Performance comparison of catalysts for photo-thermal dry reforming of methane using different values of flow rates (GHSV). – the value of light intensity is not available.

Catalyst	Temperature °C	Light Intensity mW·cm^−2^	GHSV h^−1^	Catalytic Performances	References
Ru/SrTiO_3_	600	–	4592	r(H_2_) = 320 mmol·g^−1^·h^−1^ r(CO) = 387 mmol·g^−1^·h^−1^ H_2_/CO = 0.83	[32]
Pt/TiO_2_	500	3.22	40,000	r(H_2_) = 211 mmol·g^−1^·h^−1^ r(CO) = 309 mmol·g^−1^·h^−1^ H_2_/CO = 0.68	[104]
Pt/P25	500	3.22	40,000	r(H_2_) = 40 mmol·g^−1^·h^−1^ r(CO) = 106 mmol·g^−1^·h^−1^ H_2_/CO = 0.38	[104]
Pt/TiO_2_	500	4.67	320,000	r (H_2_) = 55 mmol·g^−1^·h^−1^ r (CO) = 73 mmol·g^−1^·h^−1^ H_2_/CO = 0.75	[58]
